# Characterization of *Leiurus abdullahbayrami* (Scorpiones: Buthidae) venom: peptide profile, cytotoxicity and antimicrobial activity

**DOI:** 10.1186/1678-9199-20-48

**Published:** 2014-11-03

**Authors:** Efe Erdeş, Tuğba Somay Doğan, İlhan Coşar, Tarık Danışman, Kadir Boğaç Kunt, Tamay Şeker, Meral Yücel, Can Özen

**Affiliations:** Department of Biotechnology, Graduate School of Natural and Applied Sciences, METU, Ankara, 06800 Turkey; Molecular Biology and Biotechnology Research and Development Center, Central Laboratory, METU, Ankara, Turkey; Department of Biology, Faculty of Science, Hecettepe University, Ankara, Turkey; Department of Biology, Faculty of Arts and Sciences, Kirikkale University, Kirikkale, Turkey; Department of Biology, Faculty of Science, Anadolu University, Eskisehir, Turkey; Department of Biological Sciences, Faculty of Arts and Sciences, METU, Ankara, Turkey; Center of Excellence in Biomaterials and Tissue Engineering, METU, Ankara, Turkey

**Keywords:** Scorpion venom, Toxin, Peptide, *Leiurus abdullahbayrami*, Microfluidic capillary, Electrophoresis, Peptidomics, Venomics, Cytotoxicity, Antimicrobial activity, Turkey

## Abstract

**Background:**

Scorpion venoms are rich bioactive peptide libraries that offer promising molecules that may lead to the discovery and development of new drugs. *Leiurus abdullahbayrami* produces one of the most potent venoms among Turkish scorpions that provokes severe symptoms in envenomated victims.

**Methods:**

In the present study, the peptide profile of the venom was investigated by electrophoretic methods, size-exclusion and reversed-phase chromatography and mass spectroscopy. Cytotoxic and antimicrobial effects were evaluated on a breast cancer cell line (MCF-7) and various bacterial and fungal species.

**Results:**

Proteins make up approximately half of the dry weight of *L. abdullahbayrami* crude venom. Microfluidic capillary electrophoresis indicated the presence of 6 to 7 kDa peptides and proved to be a highly practical peptidomics tool with better resolution when compared to conventional polyacrylamide gel electrophoresis. Mass spectroscopy analysis helped us to identify 45 unique peptide masses between 1 to 7 kDa with a bimodal mass distribution peaking between molecular weights of 1 to 2 kDa (29%) and 3 to 4 kDa (31%). *L. abdullahbayrami* crude venom had a proliferative effect on MCF-7 cells, which may be explained by the high concentration of polyamines as well as potassium and calcium ions in the arachnid venoms. Antimicrobial effect was stronger on gram-negative bacteria.

**Conclusions:**

This work represents the first peptidomic characterization of *L. abdullahbayrami* venom. Considering the molecular weight-function relationship of previously identified venom peptides, future bioactivity studies may lead to the discovery of novel potassium and chloride ion channel inhibitors as well as new antimicrobial peptides from *L. abdullahbayrami* venom.

**Electronic supplementary material:**

The online version of this article (doi:10.1186/1678-9199-20-48) contains supplementary material, which is available to authorized users.

## Background

Animal venoms compose a complex mixture of ions, small organic molecules, peptides and proteins, which evolved through millions of years of natural selection aiming at prey capture and defense mechanisms. A significant part of this rich mixture is composed of bioactive peptides. Due to their remarkable structural and functional variety, these bioactive peptides offer almost limitless possibilities for the development of new therapeutic agents [1, 2].

Arachnid species including scorpions and spiders are of great interest for bioactive peptide research since the complex venom of these animals contain many peptide toxins [3]. Scorpion venoms are composed of inorganic salts, free amino acids, nucleotides, biogenic amines, peptides and proteins [4]. Peptide neurotoxins form most of their venom that may contain more than a hundred peptides [5]. So far, more than 600 scorpion peptides were described in the UniProt database [6]. These toxins were functionally classified into four groups as Na^+^, K^+^, Ca^2+^ and Cl^−^ ion channel inhibitors [7]. During 400 million years of evolution, these toxins gained excellent specificity and affinity for their targets, which makes them highly potent inhibitors [8].

According to latest records, Turkey has 23 species of scorpions in 11 *genera*. So far, venom characterization of only three buthid species was carried out. A Na^+^ channel α-toxin Bu1 was discovered from the venom of *Buthacus macrocentrus* [9]. Three K^+^ channel inhibitors MegKTx1, MegKTx2 and MegKTx3 were isolated from the venom of *Mesobuthus gibbosus* [10]. One of the two scorpion species of Turkey that may cause lethal poisoning in humans is *Androctonus crassicauda*. Biochemical characterization of its venom is in progress and recently bioactive peptides Acra1, Acra2, Acra3 and Acra4 were isolated from the venom [11–13].

The *Leiurus* genus of Buthidae family includes five species: *L. quinquestriatus*, *L. jordanensis, L. savanicola*, *L. nasheri* and *L. abdullahbayrami. Leiurus quinquestriatus* is the best characterized member of the genus from which many well-known peptide toxins such as chlorotoxin and charybdotoxin were isolated [14, 15].

**Figure 1:**
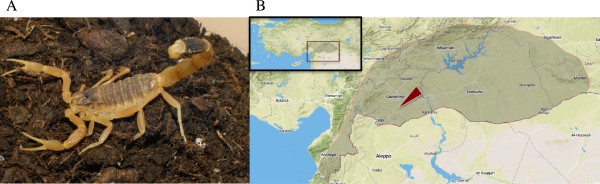
***Leiurus abdullahbayrami*** **in captivity (A) and its distribution in Turkey (B) represented by the dark area.** Red arrow shows location where the specimens were collected (http://www.mapbox.com).

*L. abdullahbayrami* (Figure 1 – A) is a recently described species of *Leiurus* genus which was previously identified as *L. quinquestriatus*. It is distributed around southeastern Turkey, mainly western Euphrates basin and specimens were recorded in Adiyaman, Hatay, Kilis, Gaziantep, Sanliurfa, and Kahramanmaras provinces (Figure 1 – B) [16]. Electrophoretic profile of its venom indicated the strong expression of 4 and 6 kDa peptides. It was also shown that *in vivo* toxicity of its venom (LD_50_) was 0.19 mg/kg on mice [17].

The aim of this study was to determine the peptide profile, cytotoxicity and antimicrobial effect of *L. abdullahbayrami* venom. Characterization studies included protein content determination, comparative electrophoretic profiling and peptide mass determination. A combination of size-exclusion and reversed-phase chromatography (RPC) was used to focus on the peptide fraction of the crude venom. Bioactivity screening was carried out on a mammalian cell line (MCF-7) and a few selected bacterial and fungal species.

## Methods

### Specimen collection and venom milking

*L. abdullahbayrami* scorpions were collected from a semi-arid steppe area of Sinanköy Village, Gaziantep Province, southeast of Turkey (37^ο^2’16”N 37°35’58”E). Specimens were maintained in plastic boxes and fed mealworms twice a month. Specimens (six males and three females, adults) were milked by electrical stimulation (15 V) of the telson. Venom samples were collected in polypropylene tubes, diluted with double distilled water and centrifuged at 15,000 g for 15 minutes at 4°C. The supernatant was transferred to new tubes, lyophilized and stored at −80°C.

### Protein content determination

Protein content of the crude venom was determined using Bio-Rad Quick Start™ Bradford Protein Assay (USA).

### Electrophoresis

Bio-Rad Mini-Protean® Tetra Cell system and microfluidic capillary electrophoresis (MCE) with Agilent Protein 80 kit on an Agilent Bioanalyzer 2100 system (USA) were used. Tris-Glycine sodium dodecyl sulfate-polyacrylamide gel electrophoresis (SDS-PAGE) was performed using 4% stacking and 10% resolving gels under denaturing conditions at constant current (25 mA). For Tris-Tricine SDS-PAGE, 4% stacking, 10% spacer and 16% resolving gels with 6 M urea were used with 35 mA [18]. Protein bands were stained by silver staining; whereas 5 μg of sample was loaded per well for Tris-Glycine and Tris-Tricine SDS-PAGE; and 0.1 μg of sample was loaded per well in MCE.

### HPLC Fractionation

A Varian Prostar high-performance liquid chromatography (HPLC) system (USA) was used for venom fractionation. First, 50 μL of crude venom (4 mg/mL) dissolved in a running buffer (10% acetonitrile and 0.1% trifluoroacetic acid in deionized water) was applied to a Tosoh Bioscience TSKgel® G2000SW size exclusion column (Japan, 7.5 mm × 600 mm, 12.5 nm pore size). Total run time was 60 minutes with 0.5 mL/minute flow rate. Peptide fraction from size exclusion chromatography (SEC) run was collected, freeze dried and dissolved in buffer A (0.1% trifluoroacetic acid in deionized water). Then, 50 μL of resuspended sample (30 μg/mL) was apllied to a Vydac® (USA) 218TP54 C18 reversed-phase column (4.6 mm × 250 mm, 300 Å pore size). Peptide fractions were eluted at 0.7 mL/minute flow rate by a linear gradient of buffer A to 60% buffer B (0.1% trifluoroacetic acid in acetonitrile) over 90 minutes.

### Mass spectroscopy

Agilent 1200 HPLC coupled LC/MS TOF 6530 mass spectroscopy system was used for the determination of molecular weight of venom peptides. Freeze dried peptide fraction from the SEC run was dissolved in buffer A (0.1% formic acid in deionized water) and 50 μL resuspended sample (30 μg/mL) was applied to an Agilent Technologies ZORBAX Eclipse XDB C18 column (4.6 mm × 150 mm, 5 μm). A linear gradient from buffer A to 60% buffer B (0.1% formic acid in acetonitrile) at 0.7 mL/minute over 90 minutes was employed. Ionization was achieved with an electrospray ionization (ESI) module followed by mass detection by TOF detector that was operating at positive ion mode with 2000 V and 100–3200 m/z range. Data analysis was performed using Agilent MassHunter Workstation Qualitative Analysis software.

### Cytotoxicity assay

MCF-7 human epithelial breast adenocarcinoma cell line was cultured in 4500 mg/L of glucose containing Dulbecco’s modified Eagle’s medium (DMEM) with 10% FBS, 2 mM L-Glutamine and 100 units/mL of penicillin-streptomycin at 37°C and 5% CO_2_. Then, 2 × 10^3^ cells were seeded in 96-well microplate and incubated for 24 hours before treatment. Crude venom (200 μg/mL) and etoposide (60 μM) were applied (24 and 48 hours) in serum-free medium to avoid interference of serum proteins. Cell viability was determined using an XTT Cell Viability kit from Cell Signaling Technology (USA). The experiments were conducted in triplicates and statistical significance of the observed difference was determined using one-way ANOVA with Tukey’s multiple comparison test of the GraphPad Prism software (USA).

### Antimicrobial activity assay

*Listeria monocytogenes*, *Escherichia coli*, *Enterobacter aerogenes*, *Pseudomonas aeruginosa*, *Candida krusei* and *Candida albicans* were obtained from the Laboratory of Microbiology, Medical Faculty, Kırıkkale University. Antibacterial activity of the venom was assessed by agar disc diffusion assay. Microorganisms were activated by inoculating a loop of the strain in the nutrient broth and incubated on rotary shaker. Then, 0.2 mL of inoculum (10^7^-10^8^ mL as per McFarland standard) was added to the Mueller Hinton agar media. Subsequently, 40 μL of venom at a concentration of 20 mg/mL was applied on the disc (0.6 cm). After 18–24 ± 2 hours of incubation at 37 ± 0.1°C, microbial growth was determined by measuring the diameter of the inhibition zone. For antifungal activity investigation, yeasts (0.5-2.5 × 10^6^/mL) were cultivated on Sabouraud 2%-dextrose agar. Venom solution was applied as mentioned above. After cultivation for 24–37 hours at 25 ± 2°C, the growth was determined by measuring the diameter of the inhibition zone. Standard antibiotic discs (RA5, TE30, AMC30 and E15) were used as positive controls. Phosphate buffered saline (PBS) soaked disks were used as negative control.

## Ethics statement

This work required no approval by Middle East Technical University Local Ethical Committee for Animal Experiments based on the reason that invertebrate animals were used in the study. All precautions were taken to ensure no harm was inflicted on the scorpion specimens during venom milking.

## Results

### Protein content and electrophoretic profile

Protein content of *L. abdullahbayrami* venom was determined by Bradford assay to be 54%. The venom of closely related member of the same genus, *L. quinquestriatus,* was previously determined to have 65% protein in its composition [19].

**Figure 2:**
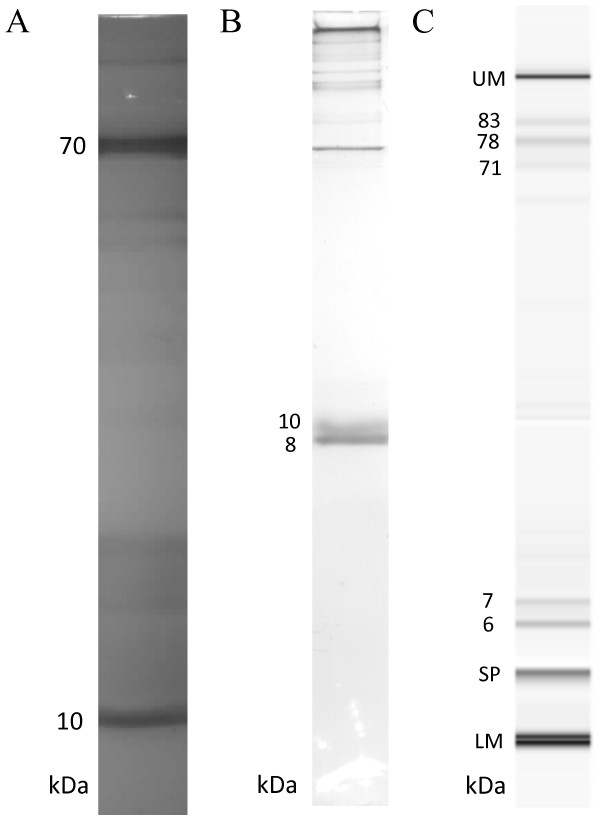
**Electrophoretic profile of** ***L. abdullahbayrami*** **venom. (A)** Glycine SDS-PAGE; **(B)** Tricine SDS-PAGE and **(C)** MCE results. UM, LM and SP stand for upper marker, lower marker and system peak, respectively.

Protein profile of the venom is showed in Figure 2. First, we analyzed venom proteins with 10% glycine SDS-PAGE which allowed us to see both high- and low-molecular-weight proteins on the same gel. Multiple proteins between 10 to 150 kDa were detected with two major bands at 10 and 70 kDa (Figure 2 – A). Since our major focus was on venomic peptides, we also used tricine SDS-PAGE, which is an electrophoretic method optimized for peptide analysis [18]. As seen in Figure 2 – B, tricine method resolves the 10 kDa glycine SDS-PAGE band into 8 and 10 kDa peptides. Proteins larger than 40 kDa could not enter the 16% resolving part of the gel as expected. As a final electrophoretic approach, we also employed MCE and detected 6 and 7 kDa peptides in addition to multiple proteins peaks in 70–85 kDa range (Figure 2 – C).

### Size exclusion chromatography

**Figure 3:**
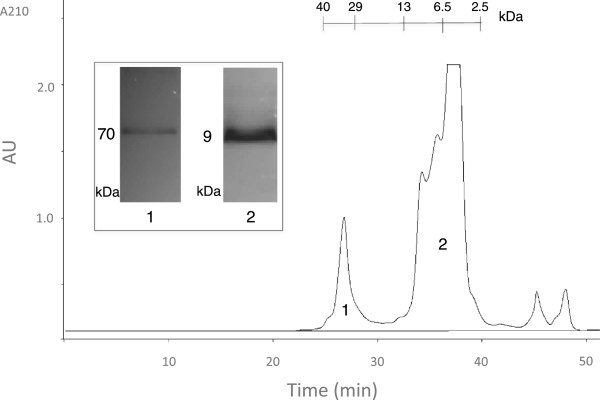
**Size exclusion chromatogram of** ***L. abdullahbayrami*** **venom.** Retention times of protein standards are shown on top. Inset shows the glycine SDS-PAGE profiles of the selected fractions.

In order to reduce venom complexity by eliminating small molecular compounds (<1 kDa) and high-molecular weight proteins (>10 kDa), we first applied the crude venom to size exclusion chromatography (SEC). As seen in Figure 3, two major fractions were obtained from this run: a relatively sharp protein peak with an estimated molecular weight of 36 kDa and a combination of multiple peptide peaks in approximately 4–10 kDa range. Surprisingly, the first SEC peak produced a single protein band of 70 kDa on the glycine SDS-PAGE. This significant molecular weight mismatch between SEC and PAGE experiments may be due to the highly compact globular structure of the protein causing retention in SEC matrix and delayed elution from the column. SEC fraction 2, which will be referred to as the peptide fraction from this point forward, was also analyzed by glycine SDS-PAGE and produced a single peptide band of 9 kDa.

### Reversed-phase chromatography

**Figure 4:**
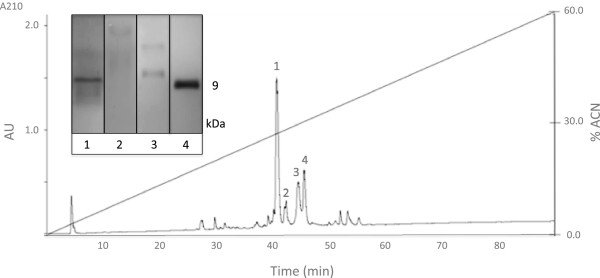
**Reversed-phase chromatogram of** ***L. abdullahbayrami*** **venom peptide fraction.** The inset shows the glycine SDS-PAGE profile of the selected peaks.

Separation of SEC fraction peptides based on their polarity was achieved by reversed-phase chromatography (RPC) (Figure 4). Since the venom complexity was reduced by a previous SEC run, a relatively clean reversed-phase chromatogram was obtained. As shown in Figure 4, four major peaks were selected and loaded to glycine SDS-PAGE to confirm their peptide identity. As expected, a 9-kDa peptide band was observed in most samples.

### Peptide mass determination

**Figure 5:**
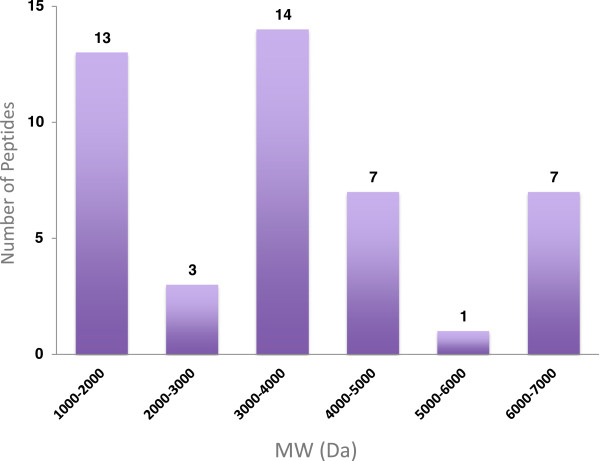
**Molecular weight distribution of** ***L. abdullahbayrami*** **venom peptides.**

For molecular weight determination, the peptide fraction of the venom obtained from the SEC column was analyzed by liquid chromatography coupled to mass spectroscopy. Table 1 summarizes the deconvoluted molecular weights of 45 peptides identified from *L. abdullahbayrami* venom*.* The mass range of the peptides was between 1032 and 6895 Da. Molecular weight distribution histogram shows a bimodal characteristic (Figure 5).

**Table 1:** **Retention times (RT) and deconvoluted molecular weights (MW) of** ***L. abdullahbayrami*** **venom peptides determined by LC-ESI-TOF – 45 molecular masses were identified**

RT (min)	MW (Da)	RT (min)	MW (Da)
11.50	2961, 3000	29.14	4540, 6810
12.40	3024	30.00	5376
12.86	2948, 2988, 3183	33.32	3555, 3576, 3591, 3615, 3630
15.39	3198	33.39	1067
15.49	3234	34.65	1155
20.90	3768	36.87	1287
21.40	3772	36.95	1331, 1375
22.56	3996	37.67	1419
23.37	4056, 4092, 4168	38.20	1463
23.79	3996, 4000	39.71	1507
24.19	4036	43.05	1032
24.86	4056	64.08	1294, 1338
25.66	6780	64.15	1426
26.62	6805, 6820, 6840, 6855, 6895	67.75	1082

### Cytotoxicity

**Figure 6:**
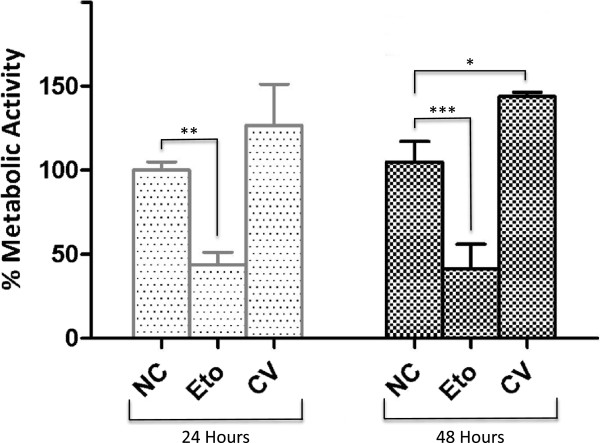
**Cytotoxicity assay of** ***L. abdullahbayrami*** **venom on MCF-7 breast adenocarcinoma cell line.** NC: negative control; Eto: etoposide (60 μM ) positive control; CV: crude venom (200 μg/mL). Error bars represent SD (*n = 3*). Stars (*, **, ***) denote statistically significant differences at *p* ≤0.05, *p* ≤0.01 and *p* ≤0.001, respectively.

Effect of *L. abdullahbayrami* crude venom on cell viability of MCF-7 human epithelial breast adenocarcinoma cell line was tested using XTT assay. As seen in Figure 6, venom (200 μg/mL) had no cytotoxic effect on the cells following 24-hour and 48-hour treatments. On the other hand, venom treatment caused a significant increase in the metabolic activity after 48 hours. This increase strongly suggests a proliferative effect of the venom.

### Antimicrobial activity

We also tested the antimicrobial activity of the venom on gram-negative (*Escherichia coli*, *Enterobacter aerogenes* and *Pseudomonas aeruginosa*) and gram-positive (*Listeria monocytogenes*) bacteria as well as two fungal species (*Candida krusei* and *Candida albicans*) using agar disc diffusion assay. Zone of inhibition values (mm) after 24 hours of treatment are provided in Table 2. Antimicrobial effect of the venom seems to be stronger on the gram-negative bacteria.

**Table 2:** **Antimicrobial activity of** ***L. abdullahbayrami*** **crude venom determined by agar disk diffusion assay**

	Zone of Inhibition (mm)	
	Crude venom	Antibiotic
*Escherichia coli*	27	20 (RA5)
*Enterobacter aerogenes*	30	37 (TE30)
*Pseudomonas aeruginosa*	20	28 (AMC30)
*Listeria monocytogenes*	16	26 (E15)
*Candida krusei*	20	21 (E15)
*Candida albicans*	23	33 (AMC30)

RA5: 5 μg of rifampin, TE30: 30 μg of tetracycline, AMC30: 30 μg of amoxicillin-clavulanic acid, E15: 15 μg of erythromycin.

## Discussion

### Microfluidic capillary electrophoresis offers significant resolution advantage

Peptidomics approach followed in this work has some differences when compared to commonly used crude venom characterization studies. In the literature, crude venom is typically separated by RPC that produces more peaks. Since our focus was on peptide components of the venom, we preferred to use SEC to separate the peptide fraction of the venom (1–10 kDa) from low- (<1 kDa) and high-molecular-weight components (>10 kDa) as suggested by Vassilevski *et al.* [20]. The peptide fraction obtained by SEC was then loaded on a C18 RPC column to separate peptides in the mix based on their polarity. This produced a clean HPLC chromatogram, which makes fraction collection and peptide bioactivity screening easier (Figure 4).

We also compared the performance of two electrophoretic methods, namely PAGE and MCE. In terms of resolution, MCE has a clear advantage over PAGE. MCE peptide bands at 6 and 7 kDa have a perfect match to MS determined peptide masses in 6–7 kDa range. Also, from a practical point of view, running an MCE experiment is significantly easier and shorter on Agilent 2100 MCE system. The major drawback of the platform was the lack of a suitable protein analysis kit for the detection of peptides below 5 kDa. Due to this limitation, we could not detect 1 to 5 kDa peptides, which were identified by MS.

We detected a major 9-kDa band in tricine SDS-PAGE runs of SEC and RP-HPLC fractions. Since MS analysis does not indicate the presence of peptides with this molecular weight, this must be due to the relatively poor resolution of our PAGE setup. Therefore what we determined as 9 kDa on tricine SDS-PAGE must be 6–7 kDa peptides actually. Lack of 3–4 kDa peptide bands on our PAGE results was quite unexpected since 3.4 and 5 kDa bands can be clearly seen in the marker lanes (Additional file 1). Since Ozkan *et al.* [17] previously showed the presence of 4-kDa *L. abdullahbayrami* peptides on glycine SDS-PAGE, we suspect that dimer formation due to the intermolecular disulfide bridges may be responsible for the lack of the expected peptide band. On the other hand, increasing the concentration and type of the reducing agent and increased duration of reduction did not change the results. This leaves the sensitivity problem due to the low abundance of 3–4 kDa peptides in the venom as the best explanation for the observed outcome.

### Most *L. abdullahbayrami*venom peptides are in the 1–4 kDa mass range

Molecular weight histogram provided in the excellent review by King and Hardy [6] indicates a bimodal mass distribution for the scorpion venom where the majority of the peptides fall in either 3.5 to 4.0 or 6.5 to 7.5 kDa ranges. Peptide mass profile of *L. abdullahbayrami* partially matches this general trend considering that one third of its peptides are in the 3–4 kDa range. However, instead of a major 6.5-7.5 kDa distribution, 30% of *L. abdullahbayrami* venom peptides fall in the 1–2 kDa range. In the light of previous peptide bioactivity studies, 3–4 kDa peptides of the venom are expected to be potassium and chloride ion channel blockers while 1–2 kDa peptides may be non-disulfide-bridged peptides that typically show antimicrobial activity [21].

### *L. abdullahbayrami*venom shows proliferative effect

*L. abdullahbayrami* crude venom did not show cytotoxic effect on MCF-7 cells at 200 μg/mL. Similarly, crude venom (250 μg/mL) of another Turkish scorpion, *Androctonus crassicauda*, has also been shown to be not toxic to various mammalian cells lines [12]. Interestingly, *L. abdullahbayrami* venom has a clear proliferative effect on the cells especially after a 48-hour treatment. This may be due to several reasons. Arachnid venoms are rich in polyamines and ions, especially potassium and calcium [22]. These low-molecular-weight components and ions have been previously shown to induce cell proliferation [23]. In addition, amino acids and proteins of the crude venom might have provided a better growth medium for the MCF-7 cells considering the serum-free medium of the control cells. Sherif *et al.* [24] has also reported a similar mitogenic effect of *L. quinquestriatus* venom fractions on Vero and BGM cells and concluded that increased calcium ion influx or interleukin and kinin-linked mechanisms might have stimulated the cell proliferation. Similarly, Heinen *et al.* [25] also observed that *Lonomia obliqua* caterpillar venom led to decrease in the production of nitric oxide and increased the viability of U138-MG and HT-29 cell lines. They also showed that activation of the cAMP signaling pathway inhibited the effects of the venom, suggesting an interesting link between venom, nitric oxide and cAMP signaling [25].

### Antimicrobial activity of the venom

Antimicrobial potency of the venom is comparable to previous studies conducted on *L. quinquestriatus* [26]. We identified 13 venom peptides with 1–2 kDa in *L. abdullahbayrami* venom by mass spectroscopy and as discussed earlier, these small peptides are typically categorized as non-disulfide-bridged peptides with antimicrobial activity.

## Conclusions

This work is the first detailed bioactivity study that used peptidomics methods for *L. abdullahbayrami* scorpion venom. Protein content of the crude venom was determined as 54%. We showed that MCE is a practical peptidomics tool with high resolution for venom research. A total of 45 venom peptide masses were identified by mass spectroscopy in the present study, 60% of which are within 1–4 kDa range. *L. abdullahbayrami* crude venom has a proliferative effect on MCF-7 tumor cell line. Various venom components including potassium and calcium ions as well as polyamines, amino acids and proteins may play a role in this outcome. We also report antimicrobial effect of the venom, which is stronger on gram-negative bacteria. Future studies on *L. abdullahbayrami* venom peptides may lead to the discovery of novel potassium and chloride channel blockers as well as antimicrobial peptides.

## Electronic supplementary material

Additional file 1: **Tricine SDS-PAGE profile of fraction number 3 and 4 of RP-HPLC (lanes 1 and 2) and protein ladder (lane 3).** As seen from the protein ladder, tricine SDS-PAGE can resolve peptides in 3.4 to 10 kDa mass range. (PPTX 79 KB)

## Abbreviations

MCE: Microfluidic capillary electrophoresis
MW: Molecular weight
RPC: Reversed-phase chromatography
SDS-PAGE: Sodium dodecyl sulfate polyacrylamide gel electrophoresis
SEC: Size exclusion chromatography.

## References

[1] Lewis RJ, Garcia ML (2003). Therapeutic potential of venom peptides. Nat Rev Drug Discov.

[2] Escoubas P, Rash L (2004). Tarantulas: eight-legged pharmacists and combinatorial chemists. Toxicon.

[3] Possani LD, de la Vega RC R, Kastin AJ (2006). Scorpion venom peptides. Handbook of biologically active peptides. 1° edition.

[4] Quintero-Hernández V, Jiménez-Vargas JM, Gurrola GB, Valdivia HH, Possani LD (2013). Scorpion venom components that affect ion-channels function. Toxicon.

[5] Delepierre M, Prochnicka-Chalufour A, Boisbouvier J, Possani LD (1999). Pi7, an orphan peptide from the scorpion *Pandinus imperator*: a 1H-NMR analysis using a nano-NMR Probe. Biochemistry.

[6] King GF, Hardy MC (2013). Spider-venom peptides: structure, pharmacology, and potential for control of insect pests. Annu Rev Entomol.

[7] Srinivasan KN, Gopalakrishnakone P, Tan PT, Chew KC, Cheng B, Kini RM, Koh JL, Seah SH, Brusic V (2002). SCORPION, a molecular database of scorpion toxins. Toxicon.

[8] Tan PTJ, Veeramani A, Srinivasan KN, Ranganathan S, Brusic V (2006). SCORPION2: a database for structure-function analysis of scorpion toxins. Toxicon.

[9] Caliskan F, Quintero-Hernández V, Restano-Cassulini R, Batista CV, Zamudio FZ, Coronas FI, Possani LD (2012). Turkish scorpion *Buthacus macrocentrus*: general characterization of the venom and description of Bu1, a potent mammalian Na^+^-channel α-toxin. Toxicon.

[10] Diego-García E, Peigneur S, Debaveye S, Gheldof E, Tytgat J, Caliskan F (2013). Novel potassium channel blocker venom peptides from *Mesobuthus gibbosus* (Scorpiones: Buthidae). Toxicon.

[11] Caliskan F, García BI, Coronas FI, Batista CV, Zamudio FZ, Possani LD (2006). Characterization of venom components from the scorpion *Androctonus crassicauda* of Turkey: peptides and genes. Toxicon.

[12] Caliskan F, Ergene E, Sogut I, Hatipoglu I, Basalp A, Sivas H, Kanbak G (2013). Biological assays on the effects of Acra3 peptide from Turkish scorpion *Androctonus crassicauda* venom on a mouse brain tumor cell line (BC3H1) and production of specific monoclonal antibodies. Toxicon.

[13] Caliskan F, Quintero-Hernández V, Restano-Cassulini R, Coronas-Valderrama FI, Corzo G, Possani LD (2013). Molecular cloning and biochemical characterization of the first Na(+)-channel α-type toxin peptide (Acra4) from *Androctonus crassicauda* scorpion venom. Biochimie.

[14] DeBin JA, Maggio JE, Strichartz GR (1993). Purification and characterization of chlorotoxin, a chloride channel ligand from the venom of the scorpion. Am J Physiol.

[15] Miller C, Moczydlowski E, Latorre R, Phillips M (1985). Charybdotoxin, a protein inhibitor of single Ca2 + −activated K + channels from mammalian skeletal muscle. Nature.

[16] Yağmur EA, Koç H, Kunt KB (2009). Description of a new species of *Leiurus* Ehrenberg, 1828 (Scorpiones: Buthidae) from Southeastern Turkey. Euscorpius.

[17] Ozkan O, Yagmur EA, Ark M (2011). A newly described scorpion species, *Leiurus abdullahbayrami* (Scorpion: Buthidae), and the lethal potency and *in vivo* effects of its venom. J Venom Anim Toxins Incl Trop Dis.

[18] Schägger H (2006). Tricine-SDS-PAGE. Nat Protoc.

[19] Chicchi GG, Gimenez-Gallego G, Ber E, Garcia ML, Winquist R, Cascieri MA (1988). Purification and characterization of a unique, potent inhibitor of apamin binding from *Leiurus quinquestriatus hebraeus* venom. J Biol Chem.

[20] Vassilevski AA, Kozlov SA, Egorov TA, Grishin EV (2010). Purification and characterization of biologically active peptides from spider venoms. Methods Mol Biol.

[21] Almaaytah A, Albalas Q (2014). Scorpion venom peptides with no disulfide bridges: a review. Peptides.

[22] Vassilevski AA, Kozlov SA, Grishin EV (2009). Molecular diversity of spider venom. Biochemistry (Mosc).

[23] Weiger TM, Hermann A (2014). Cell proliferation, potassium channels, polyamines and their interactions: a mini review. Amino Acids.

[24] Sherif N, Abu-Sinna G, El-Ghitanyl A (2000). Effect of *Leiurus quinquestriatus* venom and venom fractions on cells cultured *in vitro*. Egyptian J Biol.

[25] Heinen TE, de Farias CB, Abujamra AL, Mendonça RZ, Roesler R, da Veiga AB (2014). Effects of *Lonomia obliqua* caterpillar venom upon the proliferation and viability of cell lines. Cytotechnology.

[26] Salama W, Geasa N (2014). Investigation of the antimicrobial and hemolytic activity of venom of some Egyptian scorpion. J Microbiol Antimicrob.

