# Supplementary figures and images for: Characterization of Leiurus abdullahbayrami (Scorpiones: Buthidae) venom: peptide profile, cytotoxicity and antimicrobial activity
Source: J Venom Anim Toxins Incl Trop Dis. 2014 Nov 3;20:48. doi: 10.1186/1678-9199-20-48 (PMC4237746; doi:10.1186/1678-9199-20-48)

## Slide 1
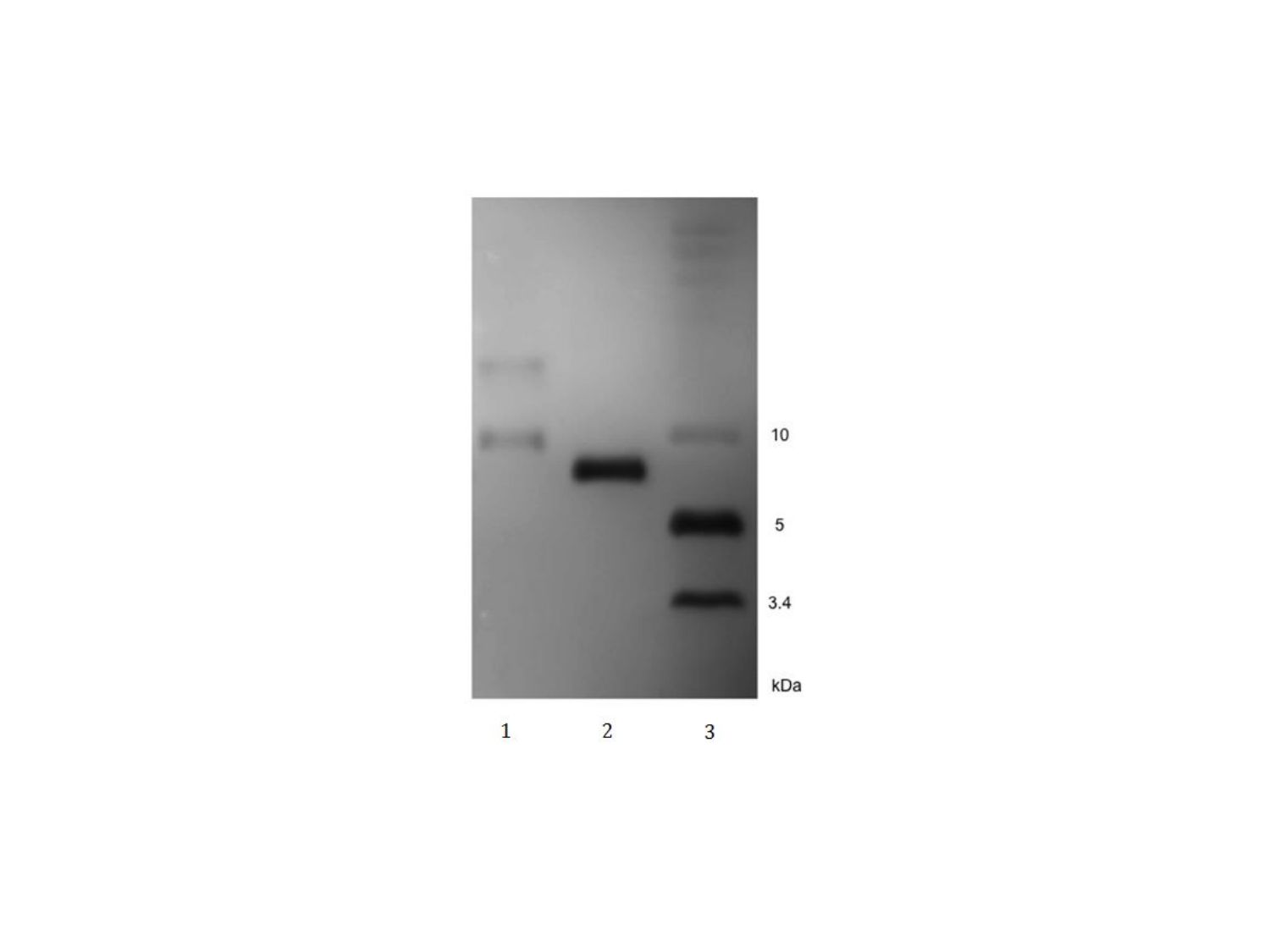

Supplement: Supplementary file 1 — Additional file 1: Tricine SDS-PAGE profile of fraction number 3 and 4 of RP-HPLC (lanes 1 and 2) and protein ladder (lane 3). As seen from the protein ladder, tricine SDS-PAGE can resolve peptides in 3.4 to 10 kDa mass range. (PPTX 79 KB) [file 40409_2014_72_MOESM1_ESM.pptx]
